# The Roles of Spinochromes in Four Shallow Water Tropical Sea Urchins and Their Potential as Bioactive Pharmacological Agents

**DOI:** 10.3390/md15060179

**Published:** 2017-06-16

**Authors:** Lola Brasseur, Elise Hennebert, Laurence Fievez, Guillaume Caulier, Fabrice Bureau, Lionel Tafforeau, Patrick Flammang, Pascal Gerbaux, Igor Eeckhaut

**Affiliations:** 1 Biology of Marine Organisms and Biomimetics Unit, Research Institute for Biosciences, University of Mons (UMONS), 23 Place du Parc, B-7000 Mons, Belgium; guillaume.caulier@umons.ac.be (G.C.); patrick.flammang@umons.ac.be (P.F.); 2 Cell Biology Unit, Research Institute for Biosciences, University of Mons (UMONS), 23 Place du Parc, B-7000 Mons, Belgium; elise.hennebert@umons.ac.be (E.H.); lionel.tafforeau@umons.ac.be (L.T.); 3 Cellular and Molecular Immunology Service, Giga Research, University of Liège (ULG), 1 Quartier HOPITAL, 11 Avenue de l’hôpital, B-4000 Liège, Belgium; laurence.fievez@ulg.ac.be (L.F.); fabrice.bureau@ulg.ac.be (F.B.); 4 Organic Synthesis and Mass Spectrometry Laboratory, Research Institute for Biosciences, University of Mons (UMONS), 23 Place du Parc, B-7000 Mons, Belgium; pascal.gerbaux@umons.ac.be

**Keywords:** spinochromes, antibacterial, antioxidant, cytotoxicity, pro-inflammatory, polyhydroxynaphthoquinones, sea urchin, pigments

## Abstract

Spinochromes are principally known to be involved in sea urchin pigmentation as well as for their potentially interesting pharmacological properties. To assess their biological role in sea urchin physiology, experiments are undertaken on crude extracts from four species and on four isolated spinochromes in order to test their antibacterial, antioxidant, inflammatory and cytotoxic activities. First, the antibacterial assays show that the use of crude extracts as representatives of antibacterial effects of spinochromes are inaccurate. The assays on purified spinochromes showed a decrease in the growth of four strains with an intensity depending on the spinochromes/bacteria system, revealing the participation of spinochromes in the defense system against microorganisms. Secondly, in the 2,2-diphenyl-1-picrylhydrazyl antioxidant assays, spinochromes show an enhanced activity compared to the positive control. This latter observation suggests their involvement in ultraviolet radiation protection. Third, spinochromes present a pro-inflammatory effect on lipopolysaccharide-stimulated macrophages, highlighting their possible implication in the sea urchin immune system. Finally, cytotoxicity assays based on Trypan blue exclusion, performed in view of their possible future applications as drugs, show a weak cytotoxicity of these compounds against human cells. In conclusion, all results confirm the implication of spinochromes in sea urchin defense mechanisms against their external environment and reveal their potential for pharmacological and agronomical industries.

## 1. Introduction

Although secondary metabolites remain ill-defined, they are commonly considered as natural and organic molecules that are not directly involved in the growth or in the reproduction of living organisms [1]. Instead, they are known to increase the survival rates of organisms producing them [1]. Secondary metabolites play various roles: they serve as defensive molecules against parasites or predators, as mediators between symbiotic organisms [2], as sexual hormones or as transporting agents [1]. Due to their interesting bioactive activities, secondary metabolites are now increasingly considered for applications in pharmacological and agrochemical industries [3] Oceans, covering 71% of the earth’s surface and being the birthplace of life, undoubtedly represent the largest potential reservoir for original and recoverable molecules [4]. Among numerous phyla occurring in marine environments, echinoderms contain many particularly interesting compounds like PolyHydroxyNaphthoQuinones (PHNQ) involved in the sea urchin’s pigmentation [4]. Also known as spinochromes or echinochromes, these molecules are present in every organ of sea urchins [5,6]). About thirty different molecular structures are already identified in the literature and are principally derivatives of polyhydroxy-1,4-naphthoquinones substituted with ethyl, acetyl, methoxy or amino groups. The most common spinochromes are named with letters (Echinochrome A and Spinochromes A–E) [4,7,8,9]. 

Although spinochromes are colored molecules, and are hence involved in the pigmentation of sea urchins, their chemical structure suggests other bioactive roles. These functions, although still poorly known, have already been investigated in several studies. Some suggest that spinochromes participate in the defense mechanisms of sea urchins by, for instance, preventing biofilm formation due to their fungicidal and antibacterial activities [10,11,12]. Their antioxidant potential and their capacity to absorb UV light—which protects sea urchins from UV-induced damages [9,13,14,15,16,17,18,19,20]—have already been reported. Some studies attribute anti-inflammatory effects to spinochromes [21,22], whereas others rather suggest their pro-inflammatory effects [23,24]. Finally, another study notes the presence of spinochromes in the gametes and embryos of sea urchins. This presence can also be linked to UV protection since it is harmful for both DNA and RNA, and so has a high importance in embryogenesis [25,26]. These bioactive effects have also been studied for medical uses. For instance, the antioxidant effects were investigated to scavenge the reactive oxygen species (ROS) involved in aging processes or in numerous different diseases like cancer, diabetes, degenerative diseases or arteriosclerosis [27,28,29]. A previous study highlighted that PHNQ from sea urchins reduces the concentration of glucose and enhances the synthesis of phospholipids in the liver [30]. For instance, Echinochrome A is commercialized as Histochrome in Russia, a drug administered against ophthalmic diseases [31] and for the prevention of myocardial infarction [15].

However, even though many studies investigated the biochemical effects of spinochromes, almost all experiments were based on crude extracts and not on isolated spinochromes [12,16,32]. In this case, it is difficult to distinguish between the real role of individual spinochromes and the role of other compounds present in extracts. For this reason, we isolated the different spinochromes’ congeners from their crude extracts, after their identification by LC-MS experiments [17,20,33,34]. To the best of our knowledge, the isolation and thus the biological assays of isolated spinochromes have not been reported previously.

The general aim of the present study is to compare the spinochromes and their bioactivities from four common regular sea urchins found on coral reef flats in the Indian Ocean: *Echinometra mathaei* (Blainville, 1825), *Diadema savignyi* (Audouin, 1809) *Tripneustes gratilla* (Linnaeus, 1758) and *Toxopneustes pileolus* (Lamarck, 1816). Although these sea urchins are found in the same habitat, they present contrasted behaviors. The burrowing sea urchin *E. mathaei* is a dark species digging itself into the basaltic and calcareous rock where it lives [35,36]. The black long-spined sea urchin *D. savignyi* is typically found well exposed on mixed sandy substrates and coral substrates [37]. The collector sea urchin *T. gratilla* is a lighter species living between coral rubble and rocks of reef flats or seagrass beds; it covers the upper surface of its body with pieces of sea grasses and seaweeds [38,39]. Finally, the flower sea urchin *T. pilleolus* is also a lighter species found in the same micro-habitats as *T. gratilla.* It also frequently covers the upper surfaces of its body but is often totally covered with small pieces of dead coral that it finds in its surroundings [40,41]. 

Crude extracts were obtained from the tests and spines of these four sea urchin species and were analyzed by mass spectrometry. The four most abundant spinochromes detected in the extracts from *E. mathaei* were then purified and their bioactive effects were compared to the effects obtained with crude extracts from the four species. Four model experiments were selected to analyze the crude extract and the spinochrome bioactivity. First, we measured the antibacterial activity. The experiments were performed on five bacterial species: two model bacteria and three marine ones known to be involved in biofilm formation [42,43,44]. Secondly, crude extracts and isolated spinochromes were tested in order to determine their antioxidant potential using the common DPPH inhibition method based on Blois’ work in 1958 [45], which consists of the scavenging capacity of antioxidants towards α, α-diphenyl-β-picrylhydrazyl (DPPH). Next, we measured the potential inflammatory impact of isolated spinochromes by the quantification of the tumor necrosis factor (TNF)-α released by lipopolysaccharide-stimulated J774 macrophages [46]. Finally, the cytotoxic effects of isolated spinochromes on HeLa cells were studied by using the Trypan Blue exclusion method, which consists of dead cell coloration by Trypan Blue dye [47,48,49].

## 2. Results

### 2.1. Extraction and Isolation of Spinochromes

Seven major spinochromes (i.e., major congeners with a concentration superior to 5% of the total PHNQ of the sea urchin species considered) were identified and quantified by mass spectrometry within all sea urchin species (Table 1): namely, Spinochrome B, two isomers of Spinochrome D, Spinochrome E, one isomer of Spinochrome A, Echinochrome A and Spinochrome C. Six other minor spinochromes were identified but will not be further discussed in the present study; a typical chromatogram can be observed in our previous study [5]. The identification of these pigments was based on the comparison of their elemental compositions based on exact mass measurements with data from the literature [4,8,20,50,51]. As identification was based on exact mass measurements; we could not determine which isomer corresponds to the Spinochrome D or A.

The comparison between the four studied sea urchin species showed that the darker sea urchins, *E. mathaei* and *D. savignyi*, presented higher PHNQ concentrations in the test and spine extracts than the two lighter sea urchins, *T. gratilla* and *T. pileolus*. *T. gratilla* contained less than 1 mG of total PHNQ by kG of dried tests/spines. *D. savignyi* contained mainly one PHNQ, Echinochrome A, and only a few of Spinochrome D – Iso 1. Although *T. pileolus* presented a large diversity of PHNQs, only Spinochrome B was present at a concentration higher than 1 mG/kG.

**Figure 1:**
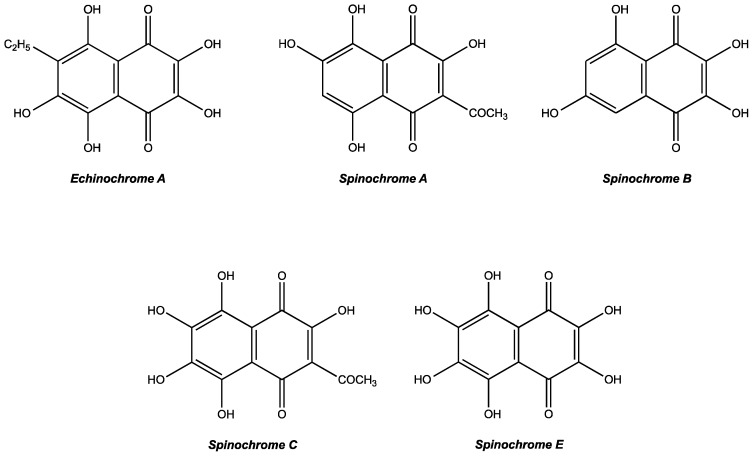
Structure of isolated *E. mathaei* tests/spines pigments collected in four fractions with the HPLC.

The isolation of spinochromes to perform the biochemical assays was carried out on the four most abundant congeners: Spinochrome A, Spinochrome B, Spinochrome E and Echinochrome A. However, Echinochrome A co-elutes with another PHNQ, Spinochrome C (equal to 75%/25% ratio). The mixture will be considered as an isolated mix. Figure 1 shows the PHNQ structures based on predicted formulae and literature confrontation.

### 2.2. Bioactive Effects of Spinochromes

#### 2.2.1. Antibacterial Activity Assay

**Figure 2:**
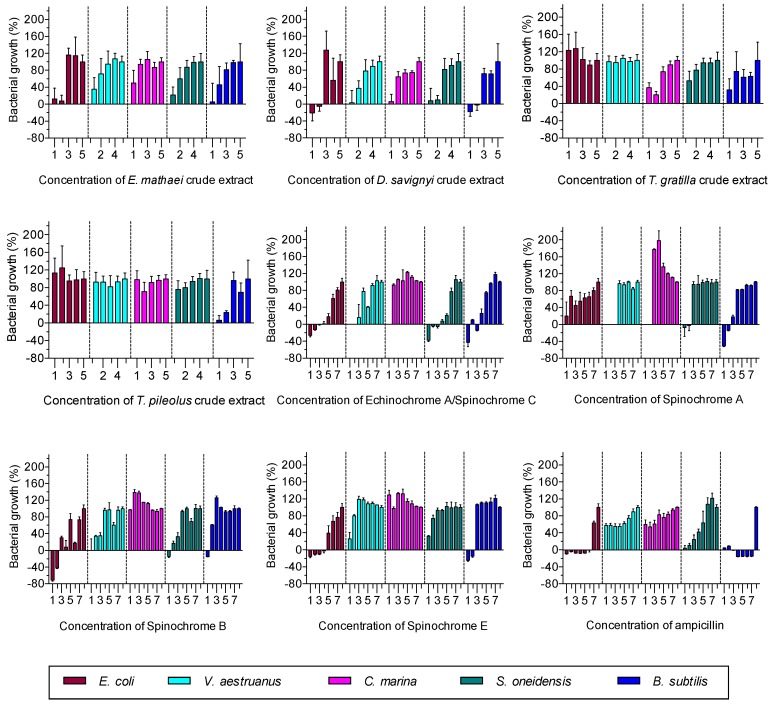
Growth (%) of bacteria incubated in medium containing sea urchin crude extracts or isolated spinochromes. Isolated spinochromes and ampicillin concentrations are expressed as 1 = 1000 μM; 2 = 500 μM; 3 = 200 μM; 4 = 100 μM; 5 = 40μM; 6 = 20 μM; 7 = 4 μM; 8 = Negative control. Crude extract concentrations are expressed as 1 = 2000 μG/mL; 2 = 1000 μG/mL; 3 = 200 μG/mL; 4 = 100 μG/mL; 5 = 1 μG/mL. Data are expressed as the mean ± one standard deviation of 3 independent experiments for each sample concentration.

The antibacterial activity of tests/spines crude extracts isolated from the four studied sea urchin species and of spinochromes isolated from *E. mathaei* extracts was tested on five bacterial strains. The effects on the bacterial growth and their calculated EC_50_ are presented in Figure 2 and Table 2, respectively.

All crude extracts presented antibacterial activity against at least one bacterial strain. *E. mathaei* crude extracts showed a strong antibacterial activity against all bacteria but appeared to be more efficient against *E. coli* and *B. subtilis* (EC_50_ < 800 μG/mL) than against the other strains (EC_50_ > 1100 μG/mL). *D. savignyi* possessed a strong antibacterial activity against all strains (EC_50_ < 700 μG/mL). *T. gratilla* was active against *B. subtilis* and had a strong activity against *C. marina* (EC_50_ < 544 μG/mL). *T. pileolus* presented a strong activity against *B. subtilis* (EC_50_ = 512 μG/mL).

Except for *C. marina* (which was not affected by ampicillin), isolated spinochromes also showed large antibacterial activity. However, several high concentrations were not tested against *V. aestruanus* and *C. marina* because of the formation of compact bacterial aggregates, making the O.D. measurements impossible. Instead, the mixed bacteria/spinochromes were incubated overnight in fresh medium in order to assess the bacterial death or survival. While the *V. aestruanus*/spinochromes mix did not show any bacterial growth at 1000 μM, the *C. marina*/spinochromes mix presented a normal bacterial growth at this dose. 

The Echinochrome A/Spinochrome C was strongly active (EC_50_ < 61 μM). Its EC_50_ was even lower than that of ampicillin against *S. oneidensis*. Spinochrome A also revealed a relatively strong activity (EC_50_ < 250 μM). Spinochromes B and E presented a more variable activity according to the bacterial strain. They showed a high antibacterial activity against *E. coli* (EC_50_ < 30 μM), but were less efficient against other strains. As a comparison, ampicillin did not possess antibacterial activity against *C. marina* and *V. aestruanus* but was strongly active against other strains.

#### 2.2.2. Antioxidant Activity Assay

**Figure 3:**
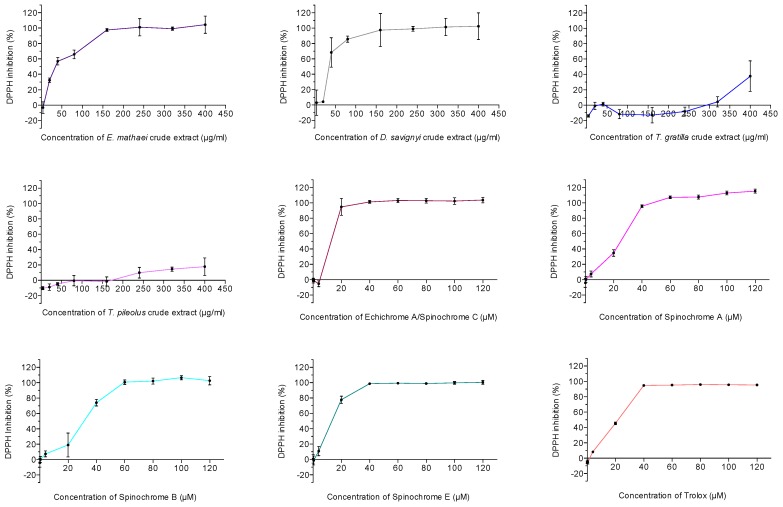
DPPH inhibition (%) of sea urchin crude extracts (*n* = 9) and isolated spinochromes (*n* = 6). Data are expressed as the means ± one standard deviation of *n* independent experiments for each concentration of a sample.

The antioxidant activities of test/spines crude extracts isolated from the four studied sea urchin species and of spinochromes isolated from *E. mathaei* extracts are shown in Figure 3. Their calculated EC_50_ are presented in Table 3. The major differences appeared between the crude extract activity among the different studied sea urchin species. *E. mathaei* and *D. savignyi* crude extracts presented largely higher antioxidant activities than those of *T. gratilla* and *T. pileolus*, with a 100% of DPPH inhibition reached around 150 μG/mL of crude extract for *E. mathaei* and *D. savignyi*, and superior to the maximal tested concentrations for *T. gratilla* and *T. pileolus*. The calculated EC_50_ of *E. mathaei* and *D. savignyi* crude extracts was around 35 μG/mL (Table 3). The EC_50_ of *T. gratilla* and *T. pileolus* crude extracts, being superior to the tested concentrations, could not be determined. On the contrary, all isolated spinochromes showed high antioxidant effects. The calculated EC_50_ were similar and even higher than the positive control, i.e., Trolox (21 μM) for Spinochrome E (11 μM) and for the Echinochrome A/Spinochrome C (16 μM). Spinochrome A and Spinochrome B EC_50_ values were quite similar to Trolox, i.e., 22 and 29 μM, respectively, and thus also presented a very high antioxidant activity. Negative values and values above 100% on figures are due to the absorbance variabilities and to the calculation method.

#### 2.2.3. Inflammatory Activity Assay

**Figure 4:**
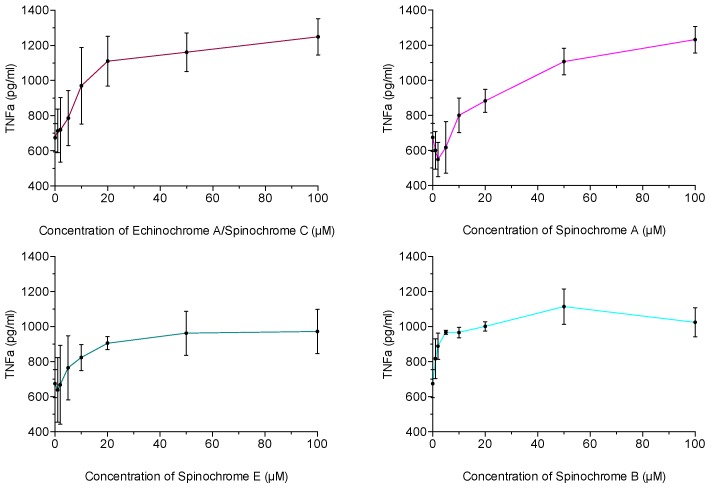
Production of TNF-α by response from J774 macrophages incubated with isolated spinochromes and stimulated with LPS. Data are expressed as the means ± one standard deviation of 3 independent experiments for each sample concentration.

The inflammatory activities of the isolated spinochromes are shown in Figure 4. Pre-incubation of J774 macrophages with each of the isolated spinochromes caused an increase in TNF-α production by these cells after LPS stimulation. Without LPS stimulation, the isolated spinochromes did not have any effect on TNF-α production (data not shown).

#### 2.2.4. Cytotoxic Activity Assay

**Figure 5:**
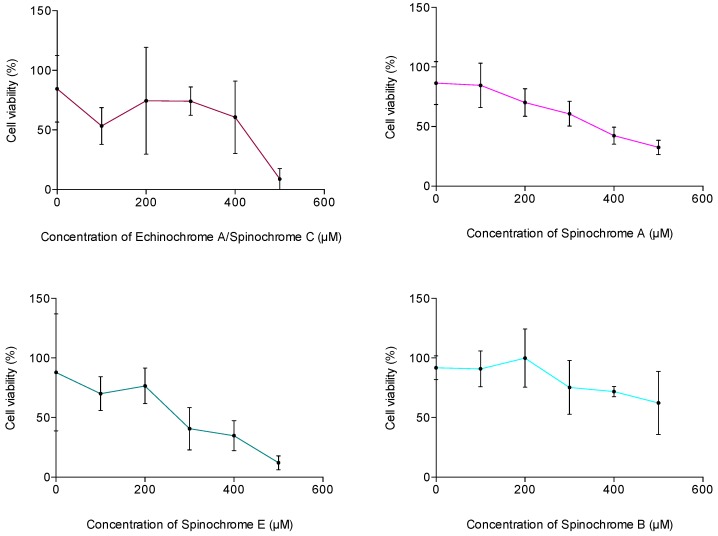
Cell viability (%) of HeLa cells incubated with increasing doses of isolated spinochromes. Data are expressed as the means ± one standard deviation of 3 independent experiments for each sample concentration.

The cytotoxic activities of spinochromes were evaluated using Trypan Blue dye, which enables dead cell coloration. The cytotoxic activities of the isolated spinochromes are shown in Figure 5. Their calculated EC_50_ values are presented in Table 4. All isolated spinochromes caused a decrease in HeLa cell viability at 500 μM. Three of them, Spinochromes A and E and the Echinochrome A/Spinochrome C mix, present a calculated EC_50_ between 259 μM and 381 μM (Table 4). The Spinochrome B EC_50_ was not calculated since the slope of the cell viability was too slight with the tested concentrations.

## 3. Discussion

The spinochromes are red to purple pigments naturally present in all body compartments of sea urchins. Several studies have already highlighted some bioactive properties, which suggest an active role of defense in sea urchins [10,51,52]. However, most of the previous research conducted on the bioactivity of spinochromes was based on crude extracts from the tests and spines of sea urchins [11,12,16,18,20,32]. The present study, comparing the bioactivity of crude extracts isolated from four studied sea urchins and spinochromes isolated from *E. mathaei*, showed that the results of bioassays can differ when using crude extracts or purified spinochromes. Therefore, it is a dangerous shortcut to discuss the role of spinochromes when using only crude extracts. This assessment is particularly evident in the species *D. savignyi*. Indeed, *D. savignyi* crude extracts showed a large antibacterial activity against all tested bacterial strains, even though these extracts only contain Echinochrome A that had no effect on *C. marina*. However, the Echinochrome A isolated does not have an antibacterial activity against *C. marina*. This antibacterial activity against *C. marina* with crude extracts from *D. savignyi* is therefore certainly due to a compound other than spinochromes or a synergic effect between Echinochrome A and another compound. 

Tropical shallow water sea urchins are preyed upon by various fishes [53,54] and parasitized by multiple organisms [55,56,57]. Diseases can also occur in sea urchins and are often induced by predators or parasites. The most common of these diseases is the bald sea urchin disease in which bacteria proliferate in wounds [58,59]. Obviously, the tests and spines of the four sea urchin species we tested presented an antibacterial activity but the extracts of two of them, *E. mathaei* and *D. savignyi*, appeared more efficient as they decreased the growth of the five tested bacteria at a concentration of 500 μG/mL while at that concentration, *T. gratilla* and *T. pileolus* crude extracts only had a significant effect on 2 and 1 bacterial species, respectively. The antibacterial assays performed with isolated spinochromes also showed contrasting results. All spinochromes had an antibacterial activity except against *C. marina* and with an effectiveness depending on the tested spinochrome and the type of bacteria. The mixed Echinochrome A and Spinochrome C had the highest activities, being effective on some bacteria already at concentration as weak as 200 μM (or 52.8 μG/mL) Spinochrome A also acted on all strains but at a higher concentration. By contrast, Spinochrome B and Spinochrome E had a lower global activity except against *E. coli.* It is important to highlight that some spinochromes were not completely soluble in water at high concentrations [60], which could lead us to underestimate some results and particularly the antimicrobial ones where high concentrations were tested.

Each spinochrome thus has a specific antibacterial spectrum that may widen the protection of some sea urchins against infections. At the study site, sea urchins are prone to infestation by the gastropod *Vexilla vexillum* that can lead to lethal bacterial infections. Out of the four studied sea urchins, only *T. gratilla* and *E. mathaei* can be infested and develop the disease, the first being 5 times more affected [61]. *D. savignyi* and *T. pileolus* are probably better protected than the two other species thanks to their efficient spines or their pedicellariae toxins [62]. Moreover, the spinochrome antibacterial pattern in *E. mathaei* may explain its better resistance compared to *T. gratilla* to bacteria after the grazing exerted by the gastropod. It was interesting to notice that spinochromes could present a higher antibacterial effect on marine strains than ampicillin: all spinochromes had a higher effect on *V. aestuarianus* while the Echinochrome A/Spinochrome C mix showed a higher effect on *S. oneidensis.* On the contrary, ampicillin stayed more efficient against model human strains. Subsequently, antibacterial activities, especially observed on marine bacteria, could lead us to consider the spinochromes as potential candidates in the field of marine aquacultures where the resistance to classical antibiotics has dramatically increased during the last years [63,64].

Reactive oxygen species (ROS) are reactive molecules containing oxygen. They are byproducts of the normal metabolism of oxygen and play important roles in cell signaling and homeostasis. With environmental stress (e.g., UV or heat exposure), ROS levels can increase dramatically and may result in significant damages to the cell structure, a phenomenon known as the oxidative stress [27,28]. Moreover, ROS are known to cause DNA damages and thus potentially affect reproduction and development [65,66,67,68]. Some studies [28,29] showed that antioxidant molecules provide protection against ROS and hence from the resulting cell damages. It is well known that spinochromes are antioxidant agents in sea urchins [9,12,14,20]. In the present study, the antioxidant properties of both crude extracts of the four studied sea urchin species and spinochromes isolated from *E. mathaei* were tested. Results based on crude extracts showed a very weak antioxidant activity for *T. gratilla* and *T. pileolus.* Such a low activity provides evidence for the absence of other antioxidant compounds in crude extracts. On the other hand, the two other sea urchins, i.e., the ones that do not show any covering behavior, showed a very high antioxidant activity. The experiments on isolated spinochromes demonstrated that Spinochrome E and Echinochrome A/Spinochrome C mix are powerful antioxidants, even more efficient than the Trolox control, an analog of vitamin E. Spinochrome antioxidant activities can be linked to their molecular structure and, in particular, to the number of their hydroxyl and acetyl groups. Spinochrome E, with the highest antioxidant activity, has 6 hydroxyl radicals. Echinochrome A and Spinochrome C, the spinochrome with the second highest antioxidant activity, both possess 5 hydroxyl radicals. They are followed by Spinochrome A with 4 hydroxyl groups and one acetyl group. Finally, Spinochrome B has only 4 hydroxyl substituents. These results are confirmed by other studies [69,70] that showed an increase in the antioxidant potential with the number of double bonds, hydroxyls as well as acetyl radicals. This hypothesis could also explain the extremely high antioxidant capacity determined for dimeric spinochromes (with 8 hydroxyl groups) as mentioned in a previous study [21]. The low spinochrome concentrations in *T. gratilla* and *T. pileolus* could explain why they cover themselves with various objects; this covering behavior could indeed overcome the lack of spinochromes and protect them against UV radiation. 

In vitro inflammatory assays led to unexpected results, in opposition with two studies where the Echinochrome A is known to present anti-inflammatory activities [22,71]. Indeed, these two studies were performed in vivo in order to treat a particular disease and then occurred in a complete metabolism. In our study, however, each isolated spinochrome significantly increased the TNF-α production by LPS-stimulated macrophages, inducing a pro-inflammatory activity. Such results were previously observed in another study, which showed the pro-inflammatory effects of crude extract from sea urchin spines [23], although authors favored the role of proteins in this pro-inflammatory activity. Another study highlighted the pro-inflammatory role of naphthoquinones in mice [72]. Moreover, these results also explain the inflammatory reactions caused by sea urchin injuries when the spine penetration in skin causes edema, erythema and granulomatous inflammation [24]. This synergy with LPS, already shown in the literature with hemoglobin [73], could contribute to inflammatory responses and then to the stimulation of the immune system. Several studies have already observed the LPS effects of sea urchin immune systems, hence showing an increase in coelomocyte recruitment (particularly red spherules cells, which contain spinochromes) and an increase in phagocytosis [74,75,76,77]. 

Based on the present analyses, we found that spinochromes have a moderate antibacterial activity, lower than the ampicillin control for human models but higher for marine ones. However, they present a high antioxidant activity, some spinochromes being even more efficient than the Trolox control. Finally, they induce a synergistic pro-inflammatory response following LPS stimulation. Cytotoxicity assays were then performed on HeLa cells to determine the impact of isolated spinochromes on human cells in view of some possible future pharmacological studies. The results show a slight decrease in cell viability at high concentrations. Spinochrome E appears to be the most active compound (EC_50_ = 259 μM, corresponding to 66 μG/mL), and Spinochrome B is the least active compound (EC_50_ > 500 μM, corresponding to 111 μG/mL). According to cytotoxicity criteria [78], only Spinochrome E is classified as a moderately cytotoxic compound (EC_50_ < 90 μG/mL). Consequently, Spinochromes A, B and the Echinochrome A/Spinochrome C mix are interesting candidates for their potential effects on human health. Future studies are necessary in order to investigate their real impacts on human cells.

In conclusion, some valuable results can be highlighted from this study. First, the use of test/spine crude extracts is not appropriate to determine the antibacterial effect of spinochromes due to the potential interference of other compounds. However, the antioxidant activities of spinochromes can be approached with crude extracts. Second, spinochromes are more concentrated in sea urchins that do not show any covering behavior, suggesting a link between the two facts. Finally, spinochromes have interesting biochemical activities—like antibacterial, antioxidant and pro-inflammatory effects—affirming their biological role in sea urchin defense mechanisms against their external environment (microorganisms, ROS or UV radiations) as well as in their immune system. If spinochromes do not appear to be highly toxic for human cells, the antioxidant activities represent a high interest in these metabolites as good candidates in pharmacology. The antibacterial activity, especially observed against marine bacteria, could also lead us to consider spinochromes as potential candidates in the field of marine aquaculture. 

## 4. Materials and Methods

### 4.1. Extraction and Isolation of Spinochromes

#### 4.1.1. Sampling

Four species of sea urchins were randomly collected by snorkeling at high tide around the coral reef of Toliara, Madagascar (23°23′34″ S, 43°38′47″ E) in November 2015: 32 *Echinometra mathaei* (Blainville, 1825), 5 *Diadema savignyi* (Audouin, 1809), 5 *Tripneustes gratilla* (Linnaeus, 1758) and 5 *Toxopneustes pileolus* were collected (Lamarck, 1816). Their tests with spines (tests/spines hereafter) were dissected not later than 10 min after sampling. They were washed in cold tap water and dried for 12 h at 90 °C in the dark (to avoid pigment deterioration) before being crushed. The tests/spines were then pooled according to species and stored in the dark at 5 °C before being extracted. Collection and sample preparation took place in the Halieutic and Marine Science Institute (University of Toliara, Madagascar), while the extraction and analyses were performed in the Biology of Marine Organisms and Biomimetics laboratory and the Organic Synthesis and Mass Spectrometry Laboratory of the University of Mons.

#### 4.1.2. Preparation of Crude Extracts from Sea Urchins

For each experiment, 5 g of crushed tests/spines was macerated in 10 mL of a 6 M HCl solution for 1 h at room temperature to remove CaCO_3_ before being filtrated under vacuum with a Buchner flask. The solution was partitioned three times against diethyl ether (*v*/*v*). The diethyl ether phases were recovered, pooled and partitioned three times against a 5% NaCl solution (*w*/*v*). Then, the ether phase was recovered and evaporated to dryness under low pressure at 60 °C using a rotary evaporator (Laborota 4001 efficient, Heidolph, Germany), re-dissolved in 1 mL of 80% methanol and centrifuged at 10,000× *g* for 10 min. Finally, the supernatant (corresponding to the “crude extract”) was recovered and evaporated to dryness using a Speed Vac (RC 10.22, VWR international, Heverlee, Belgium), weighed and stored in the dark at 5 °C before analysis. The samples were dissolved in 80% methanol before mass spectrometry analyses and in Milli-Q water for bioassays.

#### 4.1.3. Mass Spectrometry Analyses and Spinochrome Purification

For the pigment isolation, a Waters Alliance 2695 liquid chromatography device (HPLC) was used. The system comprises a quaternary pump, a vacuum degasser and an autosampler. The chromatography was performed on a reverse phase column (Kinetex^®^ 5 μm Biphenyl 100 Å, 50 × 4.6 mm, Phenomenex, Torrance, CA, USA) at 30 °C with a sample volume injection of 25 μL and a constant flow (1.25 mL/min) of a gradient of eluent A (Water, 0.1% formic acid) and eluent B (Acetonitrile) (Table 5).

The HPLC device was coupled to a mass spectrometer to allow pigment identification and to select the retention time to manually collect the pigments. The mass spectra were obtained on a Waters Quattro Ultima using an Electrospray ionization (ESI) source in the negative ionization mode by scanning between *m*/*z* 50 and 1500. The ESI conditions were as follows: capillary voltage of 3.1 kV, cone voltage of 40 V, source temperature of 120 °C and desolvation temperature of 300 °C. Dry nitrogen was used as the ESI gas with a flow rate of 50 L/h for the gas cone and 500 L/h for the desolvation gas.

To quantify the PHNQ in sea urchin tests/spines, 2-Hydroxy-1,4-naphthoquinone was chosen as an internal standard (IS). It was added to each sample at a concentration of 10 μG/mL to an 80% methanol solution. Identification and quantification were performed on crude extracts from the four species.

Accurate mass measurements allowing the prediction of the molecular formula of PHNQ ions were performed on a Waters Q-ToF Premier using ESI source, in the negative ionization mode by scanning between *m*/*z* 50 and 600 with scan durations of 1 s and an inter-scan times of 0.1 s. The ESI conditions were as follows: capillary voltage of 3.1 kV, cone voltage of 40 V, source temperature of 120 °C and desolvation temperature of 300 °C. Dry nitrogen was used as the ESI gas with a flow rate of 50 L/h for the gas cone and 600 L/h for the desolvation gas. The mass spectrometer was equipped with a lockspray source to obtain high mass accuracy for PHNQ ions. An iodide anion, [I]^-^ = 126.9045, generated from NaI aqueous solution upon ESI, was used as the lock mass. Mass spectra analyses were performed on MassLynx 4.1. mass spectrometry software (Waters, Milford, MA, USA).

As *E. mathaei* presents several highly concentrated PHNQ and could be easily collected, we selected this species for the purification of PHNQ for the biochemical tests. Four fractions of major spinochromes were collected at different times (Table 1) from numerous HPLC runs conducted with the *E. mathaei* crude extract (typical chromatogram in our previous study [5]). These 4 fractions correspond to Spinochrome E; Spinochrome B; Spinochrome A (which had a high purity) and a mix of Echinochrome A (75%)/Spinochrome C (25%), respectively. The Echinochrome A/Spinochrome C mix was constituted by two co-eluating molecules and was considered as one in our assays (molecular weight (MW) = 75% Echinochrome A + 25% Spinochrome C = 269.5 U). Each pigment fraction was evaporated to dryness using a Speed Vac (RC 10.22, VWR international) before being weighed and finally tested in each biochemical assay.

### 4.2. Bioactive Effects of Spinochromes

Antibacterial, antioxidant, inflammatory and cytotoxic activities of spinochromes were investigated to estimate their bioactive effects. In antibacterial and antioxidant assays, crude extracts from the four sea urchin species (*E. mathaei*, *D. savignyi*, *T. gratilla* and *T. pileolus*) and the four spinochromes isolated from the *E. mathaei* crude extract were tested. In inflammatory and cytotoxic assays, only the four isolated spinochromes were studied.

#### 4.2.1. Antibacterial Activity Assay

For these tests, two model bacteria and three marine bacteria involved in biofilm formation were used. The first included the gram-negative *Escherichia coli* (Ehrenberg, 1835) and the gram-positive *Bacillus subtilis* (Ehrenberg, 1835) Cohn 1872 AL. *E. coli* was cultured in Lysogeny Broth (LB) (Merck, Overijse, Belgium) at 37 °C, *B. subtilis* was cultured in CM0001 at 30 °C. The marine bacteria included: *Cobetia marina* (Cobet et al., 1970) Arahal et al., 2002 VL, *Vibrio aesturianus* Tison and Seidler, 1983 VP and *Shewanella oneidensis* Venkateswaran et al., 1999 VP. *C. marina* was cultured in CM0005 at 28 °C, *V. aesturianus* was cultured in CM0012 at 30 °C and *S. oneidensis* was cultured in CM0014 at 28 °C. All strains were purchased from BCCM collection (Brussels, Belgium). 

The assays were performed in 96-well plates by incubating 50 μL of crude extract sample (4000-2 μG/mL) or isolated spinochromes (2000-8 μM) in MilliQ water with 50 μL of suspension of an actively growing culture of bacteria. MilliQ water was used as a negative control, and ampicillin at different concentrations was used as a positive control. Blanks corresponded to the sterile culture medium mixed with the tested sample. After incubating for 4 h at adequate temperature in a shaking incubator (90 rpm), the absorbance was measured at 600 nm. Bacterial growth (%) was calculated using the following formula:

(1) $$\text{Bacterial growth }(\%)=100-\frac{A_{sample}-A_{blank}}{A_{control}-A_{blank}}\times 100$$

where ***A****_sample_* is the absorbance measured after incubation, ***A****_blank_* is the absorbance of the blank and ***A****_control_* is the absorbance of the negative control as described above.

The EC_50_ (the half maximal effective concentration), i.e., the sample concentration necessary to decrease the bacterial growth by 50%, was calculated from the results using a non-linear regression performed with the “Prism 6” (GraphPad) software (San Diego, CA, USA). The MIC (the Minimum Inhibitory Concentration), i.e., the sample concentration necessary to completely inhibit the bacterial growth, was calculated from the results using a non-linear regression based on the Lambert and Pearson method [79] and was performed with the “Prism 6” (GraphPad) software.

The effect of some high concentrations of purified spinochromes against *V. aestruanus* and *C. marina* is not shown because the bacteria/spinochrome mix formed dense clusters making the absorbance reading impossible. In these cases, after the experiment, the 100-μL mix was suspended in 20 mL of medium and incubated overnight in order to determine the viability of bacteria by absorbance measurement. 

#### 4.2.2. Antioxidant Activity Assay

The antioxidant activity was measured using the DPPH radical-scavenging capacity. This classical method is based on the antioxidant capacity to reduce a purple-colored stable free radical, 2,2-diphenyl-1-picrylhydrazyl (DPPH). This capacity measurement is based on the absorbance of DPPH becoming pale yellowish-colored after the antioxidant reduction [80]. The assays were performed in 96-well plates by mixing 50 μL of a solution of 160 μM DPPH (Sigma Aldrich, St. Louis, MO, USA) in methanol with 50 μL of the crude extract samples (800-8 μG/mL in 100% methanol– *n* = 9 for each concentration) or isolated spinochromes (240-0.08 μM in 100% methanol– *n* = 6 for each concentration). Methanol mixed with DPPH was used as a negative control, and Trolox (Sigma Aldrich), a classic antioxidant analog of vitamin E, mixed with DPPH was used as a positive control. Blanks corresponded to methanol mixed with the tested sample without the DPPH. After incubation for 30 min at room temperature, the absorbance was measured at 560 nm. The DPPH inhibition (%) was calculated using the following formula:

(2) $$\text{DPPH inhibition }(\%)=100-\frac{A_{sample}-A_{blank}}{A_{control}-A_{blank}}\times 100$$

where ***A****_sample_* is the absorbance measured after incubation, ***A****_blank_* is the absorbance of the blank as described above, and ***A****_control_* is the absorbance of the negative control. The EC_50_, i.e., the sample concentration necessary to decrease the initial DPPH concentration by 50%, was calculated from the results using a non-linear regression performed with the “Prism 6” (GraphPad) software.

#### 4.2.3. Inflammatory Activity

The inflammatory activity was evaluated by the quantification of TNF-α, an indicative cytokine for inflammatory responses, produced by J774 macrophages. J774 macrophages were cultured in Dulbecco’s Modified Eagle’s Medium (ref 41966-029; Thermofisher Scientific, Asse, Belgium) supplemented with 10% fetal bovine serum and 50 UI/mL Penicillin/Streptomycin (i.e., complete DMEM) in a humidified atmosphere at 37 °C with 5% CO_2_. They were plated at a concentration of about 2.5 × 10^5^ cells per mL in 24-well plates and allowed to grow for 24 h. The inflammatory activity was evaluated by replacing the medium by 1 mL of complete DMEM containing the different isolated spinochromes (100-1 μM) solubilized in DMEM. After a one-hour incubation period, the macrophages were stimulated with 10 ng/mL lipopolysaccharide (LPS, *Escherichia coli* O55:B5, Sigma) in order to initiate the inflammatory response, and incubated again for 6 h. Complete DMEM alone was used as a negative control. Cell-free supernatants were then harvested, centrifuged at 400× *g* for 5 min and stored at −80 °C for later TNF-α quantification. The TNF-α quantification was performed using a commercial ELISA kit according to the manufacturer’s recommendations (eBiosciences).

#### 4.2.4. Cytotoxic Activity

The cytotoxic activity was evaluated using Trypan Blue exclusion staining, which colors dead cells. HeLa cells (ATCC CCL-2) were cultured in Dulbecco’s Modified Eagle’s Medium (ref D6429; Sigma) supplemented with 100 UI/mL Penicillin, 100 μG/mL streptomycin and 2 mM glutamine (i.e., complete DMEM) in a humidified atmosphere at 37 °C with 5% CO_2_. The cells were plated at a concentration of about 5 × 10^4^ cells per mL in 96-well plates and allowed to grow for 24 h. Cell viability was evaluated by replacing the medium wit 200 μL of the isolated spinochromes (500–100 μM) solubilized in DMEM. DMEM alone was used as a negative control.

After an incubation period of 24 h, the medium was removed, and the cells were detached from the plates using 100 μL of 0.05% trypsin-0.53 mM Trypsin-EDTA (Gibco, Thermofisher Scientific, Asse, Belgium); 100 μL of a 0.4% (*w*/*v*) trypan blue solution in water was added, mixed and allowed to stand for 5 min. The cells were then counted using a Neubauer improved cell counting chamber. Then, 10 μL of dye-cell suspension was loaded in both chambers of the hemocytometer, and 5 squares of each chamber were counted under a light microscope.

The cell viability (%) was calculated using the following formula:

(3) $$\text{Cell viability }(\%)=\frac{C_{alive}}{C_{total}}\times 100$$

where ***C****_alive_* is the count of living cells (i.e., non-stained cells) and ***C****_total_* is the count of total cells of the blank (that allows taking into account dead cells eliminated due to the medium removal after 24 h of incubation). The EC_50_, i.e., the spinochrome concentration necessary to decrease the cell viability by 50%, was calculated from the results using a non-linear regression performed with the “Prism 6” (GraphPad) software.

## Figures and Tables

**Table 1:** Major PolyHydroxyNaphthoQuinones (PHNQs) molecules (i.e., representing more than 5% of the total PHNQ content in the species) detected in crude extracts from tests and spines of the four selected sea urchin species, ranked by increasing molecular weight.

PHNQ	Retention Time (min)	MW (U)	Predicted Formula ^A^	PHNQ Concentration (MG of PHNQ/KG of Dried Tests/Spines) ^B^			
				*E. mathaei*	*D. savignyi*	*T. gratilla*	*T. pileolus*
Spinochrome B	2.75	222	C_10_H_6_0_6_	9.2 ± 6.9	-	-	4.5 ± 4.4
Spinochrome D — Iso 1	2.65	238	C_10_H_6_O_7_	-	1.6 ± 1.7	-	0.6 ± 0.8
Spinochrome D — Iso 3	8.65	238	C_10_H_6_O_7_	-	-	0.1 ± 0.1	-
Spinochrome E	1.73	254	C_10_H_6_O_8_	4.9 ± 2.1	-	0.2 ± 0.2	0.4 ± 0.6
Spinochrome A — Iso 2	7.95	264	C_12_H_8_O_7_	13.0 ± 5.0	-	-	0.6 ± 0.7
Echinochrome A	6.69	266	C_12_H_10_O_7_	10.3 ± 2.2	17.6 ± 8.8	0.1 ± 0.1	0.2 ± 0.2
Spinochrome C	6.73	280	C_12_H_8_O_8_	2.4 ± 1.2	-	-	0.6 ± 0.9

^A^ Based on accurate mass measurements. ^B^ PHNQ concentrations are expressed as mean ± SD, *n* = 4.

**Table 2:** EC_50_ and MIC values for antibacterial activity of crude extracts, isolated spinochromes and ampicillin. EC_50_ and MIC values for isolated spinochromes and ampicillin are presented in μM and are converted into μG/mL in order to compare them with crude extracts. “-“ was used for spinochrome/bacteria systems where the EC_50_ or MIC value could not be determined because of bacterial aggregates.

	Antibacterial Activity						
	EC_50_ and MIC—Bacterial Growth						
	*E. coli*	*B. subtilis*	*V. aestuarianus*	*C. marina*	*S. oneidensis*		
Tests/Spines Crude Extracts (μG/mL)							
*E. mathaei*	688.40	716.70	1518.00	1995.00	1118.00	EC_50_	
	>2000	>2000	>2000	>2000	>2000	MIC	
*D. savignyi*	358.70	252.40	534.80	681.00	391.00	EC_50_	
	406.80	1219.00	>2000	>2000	>2000	MIC	
*T. gratilla*	>2000	1227.00	>2000	543.80	>2000	EC_50_	
	>2000	>2000	>2000	>2000	>2000	MIC	
*T. pileolus*	>2000	512.60	>2000	>2000	>2000	EC_50_	
	>2000	>2000	>2000	>2000	>2000	MIC	
Isolated Spinochromes (μM; μG/mL)							
Echinochrome A/Spinochrome C	22.56	60.98	-	>1000	27.88	μM	EC_50_
	6.08	16.43		>269	7.51	μG/mL	
	54.19	149.40	-	628.50	48.26	μM	MIC
	14.60	40.26		169.38	13.00	μG/mL	
Spinochrome A	199.40	139.20	-	>1000	238.20	μM	EC_50_
	52.64	36.75		>264	62.88	μG/mL	
	>1000	228.40	-	>1000	435.70	μM	MIC
	>264	60.30		>254	115.02	μG/mL	
Spinochrome B	16.22	510.50	214.70	>1000	172.40	μM	EC_50_
	3.60	113.331	47.66	>222	38.27	μG/mL	
	468.30	583.50	>1000	>1000	238.60	μM	MIC
	103.96	129.54	>222	>222	52.97	μG/mL	
Spinochrome E	28.53	295.20	746.70	>1000	742.60	μM	EC_50_
	7.25	74.98	189.66	>254	188.62	μG/mL	
	90.33	422.40	>1000	>1000	>1000	μM	MIC
	22.94	107.29	>254	>254	>254	μG/mL	
Ampicilin (μM; μG/mL)	4.26	1.31	>1000	>1000	83.55	μM	EC_50_
	1.49	0.46	>349	>349	65.82	μG/mL	
	8.10	1.80	>1000	>1000	272.00	μM	MIC
	2.83	0.63	>349	>349	94.93	μG/mL	

**Table 3:** EC_50_ values of antioxidant activity for crude extracts, isolated spinochromes and Trolox. EC_50_ values for isolated spinochromes and Trolox are presented in μM and are converted into μG/mL to compare them with crude extracts.

	Antioxidant Activity	
	EC_50_—DPPH Inhibition	
Sea Urchin Crude Extracts	(μG/mL)	
*E. mathaei*	35.63	
*D. savignyi*	34.46	
*T. gratilla*	>400	
*T. pileolus*	>400	
Isolated Spinochromes	(μM)	(μG/mL)
Echinochrome A/Spinochrome C	16.31	4.40
Spinochrome A	22.17	5.85
Spinochrome B	29.38	6.52
Spinochrome E	10.85	2.76
Trolox (μM)	20.88	5.22

**Table 4:** EC_50_ values of cytotoxic activity of isolated spinochromes against HeLa cells.

	Cytotoxic Activity
	EC_50_—Cell Viability
Isolated Spinochromes (μM)	
Echinochrome A/Spinochrome C	380.9
Spinochrome A	341.7
Spinochrome B	>500
Spinochrome E	258.6

**Table 5:** Gradient timetable used for the HPLC separation.

Time (Min)	Eluent A (%)	Eluent B (%)	Curve
00	80	20	Equilibration
00 → 15	80 → 50	20 → 50	Linear gradient
15 → 16	50 → 80	50 → 80	Linear gradient
16 → 18	80	20	Isocratic
